# Leveraging dairy cattle dietary preference for pasture diversification to optimize marginal land recruitment into agroecosystems

**DOI:** 10.3168/jdsc.2025-0958

**Published:** 2026-04-20

**Authors:** Abigale H. Zoltick, Thomas D. Parsons

**Affiliations:** 1 Center for Stewardship Agriculture and Food Security, New Bolton Center, University of Pennsylvania School of Veterinary Medicine, Kennett Square, PA 19348; 2 Department of Clinical Studies, New Bolton Center, University of Pennsylvania School of Veterinary Medicine, Kennett Square, PA 19348

## Abstract

A growing global population and increasing demand for food necessitate the efficient use of agricultural land. Marginal land accounts for nearly one-quarter of the total land area worldwide. Cattle can contribute to food security and environmental sustainability by grazing this otherwise underutilized land, thereby conserving arable land for crop production. However, strategic pasture management is essential to ensure that the nutritional needs of higher-yielding dairy cattle are met. While it is well documented that pasture diversification can provide valuable ecosystem services, there is a scarcity of research exploring plant biodiversity in the context of dairy cattle dietary preferences during marginal land restoration. In reviewing these topics at the interface of dairy farming and agroecology, inclusion eligibility was limited to dietary preference studies conducted on pastured dairy heifers or cows in temperate geographic regions. Thus, here we first describe the available scientific literature investigating dietary preference in pastured dairy cattle and the impact of pasture diversification. We then identify key knowledge gaps that will inform future research targeting sustainable dairy farming on marginal land with the goal of leveraging dairy cattle dietary preferences to maximize the benefits of pasture diversification.

## Highlights

• Marginal land accounts for nearly one-quarter of the total land area worldwide.
• Plant biodiversity increases the quality of established pasture and marginal land.
• Dairy cattle express dietary preferences that are context-dependent.
• Cows will graze diverse plant species when provided the opportunity.
• Plant species diversity offers a path for restorative use of marginal land.

## Graphical Abstract

A growing global population and increasing demand for food necessitate the efficient use of agricultural land. Cows in pasture-based dairy systems contribute to resource efficiency by restoring marginal land through grazing; however, carefully considered land management strategies are needed to support a dairy cow's nutritional requirements. Pasture diversification can enhance pasture quality and yield by increasing plant biodiversity while maintaining animal productivity. An integrated approach to pasture-based dairy farming that leverages the animal's dietary preferences with pasture diversification is expected to facilitate the conversion of marginal land to productive land. This whole-system approach that considers both the land and the grazing animal promises to improve sustainable land-use strategies.

**Figure:**
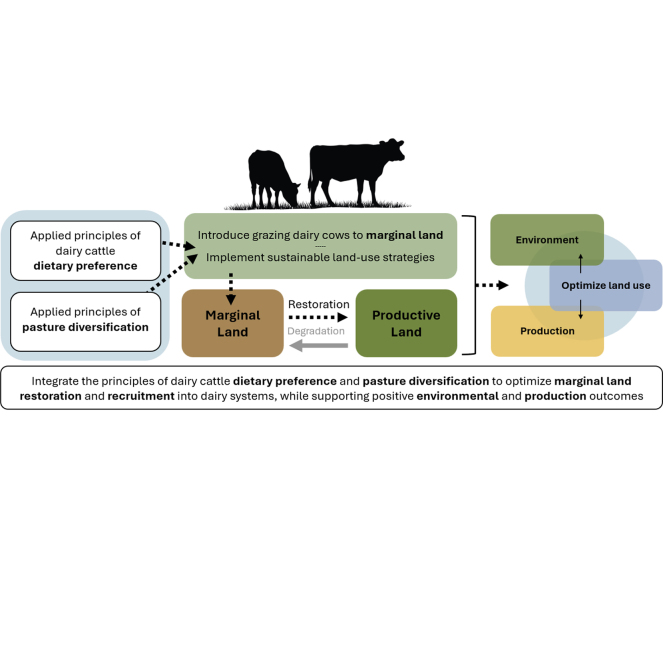

Optimizing pasture utilization by ruminants is crucial for ensuring food security, maintaining land health, and enhancing animal productivity. A growing global population and rising demand for food foretell a need for increased productivity from existing farmlands. However, the recruitment of marginal lands into productive systems offers a complementary strategy with possible added benefits. It is well documented that pasture diversification can provide valuable ecosystem services. However, there is a scarcity of research exploring plant biodiversity in the context of dairy cattle dietary preferences during marginal land restoration. To identify both controlled and observational studies on this topic, the databases consulted were Web of Science (https://www.webofscience.com/), Google Scholar (https://scholar.google.com/), and PubMed (https://pubmed.ncbi.nlm.nih.gov/), and inclusion eligibility was limited to dietary preference studies conducted on pastured dairy heifers or cows in temperate geographic regions.

Marginal land, which has limited productive potential, accounts for ~21% of the total global land area (Ahmadzai et al., 2022). Marginal land quality varies widely in terms of biophysical and economic constraints, with some land offering greater potential for regeneration and therefore greater adaptive capacity for dairy systems. Although unsuitable for crop production, ruminants can use this land due to their ability to access energy from “indigestible” forage (Csikós and Tóth, 2023). With appropriate grazing management, ruminants further demonstrate the ability to restore these degraded lands (Teague and Kreuter, 2020). However, the nutritional needs of higher-yielding dairy cattle likely require more intentional methods of land management to support their performance. These goals may be achieved through simple plant diversification or other more integrated practices, such as silvopasture—the combination of trees, forage crops, and grazing animals—that provide diverse ecosystem services.

Understanding a dairy cow's dietary “preference,” or what she chooses to eat as measured by the relative intake of feed (Oliveira et al., 2024), may inform marginal land conversion to productive pasture. Pasture diversification is a prerequisite to the expression of dietary preference and can occur in many forms, including the presence of different plant species, the same plant species at varying stages of maturity, or spatial variation of the same or different plants within a pasture. This variety leads to a greater diversity of primary and secondary biochemical compounds within the available forage, contributing to diverse smells, textures, and flavors with a range of possible physiological benefits. By understanding a cow's biologically and environmentally driven dietary preferences within the context of pasture diversification, we can implement sustainable land-use strategies that enable the cow and land to function symbiotically. The goal is not a hyperdiverse pasture that focuses on sheer species number. Understanding a cow's dietary preferences may optimize functionally diverse pasture systems that consider plant species composition, relative abundance, and specific ecological roles that support both cows and the land they graze.

Historically, spatially separated monocultures on established pastureland enabled researchers to study dietary preference in dairy cattle. Early preference research in pastured dairy cows focused primarily on spatially separated binary monocultures of perennial ryegrass and white clover with an emphasis on herd-level preferences. Rutter (2006) outlined the collective findings of this early research, reporting that cows prefer a mixed diet with a 70:30 partial preference for legumes that may vary based on life stage, time of day, and season (Cosgrove et al., 1995). Rutter et al. (2004b) reported that lactating cows have a 73.8% partial preference for clover compared with 63.9% in heifers (Rutter et al., 2004a), and both cows and heifers exhibit a stronger preference for clover in the morning.

The preference of dairy cattle for specific plant morphological and chemical characteristics has been studied by planting different cultivars of single plant species as spatially separated monocultures. Focusing solely on ryegrass, Balocchi and López (2009) found that dairy cattle spent more time grazing tetraploid versus diploid cultivars, which the researchers attributed to a higher concentration of water-soluble carbohydrates (**WSC**)—highly digestible and energy-rich sugars that support rumen microbial fermentation (Moorby et al., 2006). Smit et al. (2006) studied dairy cattle preference for 6 different perennial ryegrass cultivars with morphological and chemical differences. Researchers did not detect a significant effect of plant morphology on preference; however, chemical composition affected preference, with cows exhibiting a stronger preference for grasses with higher WSC and digestible organic matter concentrations. A later study comparing dairy cattle preference for 4 perennial ryegrass cultivars of varying sugar content found that cows preferred cultivars with greater levels of crude protein rather than those with higher concentrations of sugar (Rivero et al., 2019). These varied outcomes highlight our limited understanding of how multiple intrinsic and extrinsic factors interact to influence dietary preference.

The study of spatially distinct monocultures has its limitations, although they have served as a key experimental model for early efforts to understand dairy cattle dietary preference. It may not be feasible for a farm to plant or maintain separate monocultures in a single pasture, especially in rotational grazing systems. A possible compromise is to account for a cow's diurnal grazing preferences in a practice referred to as “temporal allocation,” which involves providing cows access to clover monoculture in the morning and grass monoculture in the evening, as reviewed by Chapman et al. (2007). This temporal pattern of grazing supports the proposed evolutionary motivation of cows to consume higher-fiber plant species in the evening, thereby reducing nighttime grazing and exposure to predators. However, there are also limitations to temporo-spatial monoculture models. When grazed in isolation, clover may increase the risk of bloat (Phillips et al., 1996), whereas mixed grass-legume pastures can offer other benefits. Clover contains a greater proportion of nitrogen relative to carbon compared with grass, and when grazed alongside grass, may enhance nitrogen fixation and optimize protein-energy balance in livestock, as reviewed by Khatiwada et al. (2020). Furthermore, although temporal allocation facilitates diurnal patterns of grazing preference, it discounts a complex array of other factors that motivate dietary preference, as well as individual variations in grazing behavior.

Breed-specific predispositions and shared evolutionary incentives associated with feeding behavior contribute to herd-level grazing patterns; however, cows are individuals with different endogenous motivating factors that shape dynamic dietary preferences. These preferences are influenced by physiological and metabolic drivers, including nutritional state, ruminal composition, age, reproductive stage, and disease state. Dominance hierarchy, personality, and early life experiences, such as food exposure, are social and biopsychological factors that also contribute to grazing behavior (Neave et al., 2018; Hesselmann et al., 2025). Dietary preferences can vary based on season and time of day (Rutter, 2006). There is also evidence that ruminants alter dietary selection to overcome parasitic infections, attenuate bloat, resolve acidosis, and self-medicate after consuming toxins (Phy and Provenza, 1998; Rutter et al., 2004a; Villalba and Provenza, 2007), consistent with the notion that animals can modulate dietary preferences based on their physiological needs. However, the field's historical focus on binary monocultures masked these individual-level variations in dietary preference by limiting available forage options.

More diversified pasture models, fueled by a growing interest in pasture biodiversity, bring to light these individual-level variations in dairy cattle dietary preference. Hesselmann et al. (2025) provided cows with continuous access to 4 plant mixtures (grasses, grasses and legumes, grasses and tanniferous plants, and grasses and herbs with essential oils). The researchers similarly reported a herd-level partial preference for the mixture containing legumes; however, the cows grazed all mixtures and exhibited individual differences in preference that varied over time. Carpino et al. (2003) studied the selective feeding behavior of dairy cows in a mixed pasture containing over 100 plant species. Although cows exhibited a strong herd-level preference for grass and legume, the researchers found that they grazed various nongrass and nonlegume plants with individual-level preference variability.

Dietary preference studies conducted on silvopasture—a deliberately diversified system—may inform marginal land conversion, as lower quality lands have successfully adapted to this land-use model (Greene et al., 2023). Vandermeulen et al. (2018) investigated dairy heifer dietary preference within a silvopastoral system that contained 11 woody browse species. The researchers reported that heifers consumed these browse species throughout the grazing season, demonstrating a greater preference in late spring when the browse had higher nutritional value despite the concurrent abundance of herbaceous forage. Consistent with dietary preference findings in lactating cows, the heifers displayed individual differences in preference for specific browse species. Collectively, these studies conducted on traditional pastureland and silvopasture corroborate earlier findings: cows demonstrate herd-level partial preferences that are subject to temporal modification. However, preferences are also influenced by individual variations and cows will graze nonlegume and nongrass species when provided the opportunity.

More diversified pasture research models have also provided greater insight into herd-level preferences and productivity, which were oversimplified in studies evaluating dairy cattle in species-limited swards. Consistent with earlier findings in studies utilizing spatially separated monocultures of clover and perennial ryegrass, Horadagoda et al. (2009) determined that dairy cows provided unrestricted access to 14 different forage species preferred white clover over perennial ryegrass. However, investigation of the additional plant species revealed that cows preferred prairie grass and kikuyu over clover. Similar to other studies that correlated plant chemical composition with preference, the researchers found a positive relationship between preference and WSC concentration. Oliveira et al. (2024) also studied herd-level dietary preference using a diversified pasture model. Cows were offered unrestricted access to binary and 4-plant-species pastures at various leaf regrowth stages, undergoing the same methods of management. The researchers reported that cows utilized both pastures and did not detect a significant difference between the amount of time spent grazing each. They concluded that, with appropriate pasture management that considers plant defoliation intervals, producers can introduce diverse plant species without negatively affecting pasture quality or feed intake.

Plant biodiversity enhances land quality and productivity in agroecosystems, with potential application to marginal lands. Finn et al. (2013) studied agricultural grassland systems comprising either monocultures or mixed plant species at 31 locations over a 3-yr period. Throughout the study, grasslands containing plant mixtures yielded higher totals and exhibited greater weed resistance than monoculture grasslands. Plant diversity also enhanced grassland resilience by mitigating stress-induced growth reductions and promoting continuous growth in the face of environmental extremes, such as drought. Even in the absence of legumes, grassland biodiversity increases carbon and nitrogen soil storage, as evidenced in an 11-yr experiment conducted by Cong et al. (2014). However, these studies compare multispecies swards with monoculture pastures rather than 2-species grass-legume pastures, which more accurately represent the current pasture paradigm. Ecosystem benefits from plant diversity may be limited in grass-legume pastures and are likely more fully realized with the establishment of agroforestry systems on degraded or marginal lands (FAO, 2017).

Studies that integrate dairy cows into multispecies pastures provide evidence that diversified grazing systems can yield environmental benefits; however, early results demonstrated equivocal animal production findings. Sanderson et al. (2005) reported increased land productivity and weed suppression in a diversified pasture system when compared with a simple binary mixture of grass and legume grazed by dairy cows. Soder et al. (2006) studied herbage yield and the performance of dairy cattle grazing 2-, 3-, 6-, and 9-species pastures. The multispecies pastures yielded greater amounts than the 2-species pasture; however, the researchers did not detect a significant effect on measures of production. These production findings align with a 2007 review concluding that early studies on plant species diversity and livestock performance demonstrate inconsistent results (Soder et al., 2007).

More recent studies demonstrate a positive impact of plant diversity on dairy cattle performance. Roca-Fernández et al. (2016) investigated environmental and animal production parameters for pastures comprised of perennial ryegrass, white clover, red clover, chicory, and tall fescue grazed as combinations of 1, 3, 4, or 5 species. The researchers concluded that multispecies pasture resulted in a greater amount of milk solids and overall milk yield, which they attributed to enhanced pasture quality and increased dry matter intake. Pembleton et al. (2016) examined the effect of various plant species on milk production at different stages of lactation. Cows were given access to one of the following: a perennial ryegrass monoculture, a white clover-plantain mixture, a perennial ryegrass-white clover-plantain mixture, or adjacent monocultures of these 3 plant species. The more diversified pastures resulted in greater milk yield during early lactation and an increase in milk protein and fat yield during mid lactation.

The benefits of plant diversity, as demonstrated by more recent studies, encompass optimized nitrogen utilization in addition to improved performance outcomes. Bryant et al. (2017) evaluated the effects of binary versus multispecies pastures on milk production and nitrogen excretion in dairy cows. Although the amount of legume present had a greater impact on milk yield than the number of plant species, the researchers determined that multispecies pasture with forbs such as plantain and chicory may reduce nitrogen losses while maintaining milk production. Carmona-Flores et al. (2020) studied 2- and 6-species pastures and examined dairy cow dry matter intake, milk yield, nitrogen partitioning, and methane emission. Cows grazing the diverse multispecies pastures demonstrated a reduction in urinary nitrogen and methane emissions. Although milk yield was not affected by treatment, cows in diverse pastures exhibited an increase in milk solids and protein production. Totty et al. (2013) studied dairy cows grazing either white clover and ryegrass, white clover and high-sugar ryegrass, or a mixed pasture containing white clover, high-sugar ryegrass, chicory, plantain, and lotus. Similar to Carmona-Flores et al. (2020), the researchers reported improved milk protein production and a reduction in urinary nitrogen with more diverse pasture. Although the findings are not uniform, these studies provide evidence that pasture diversification has the potential to improve ecosystem health and maintain or increase facets of milk production. The impact on farm profitability, however, depends on the cost structure of these plant diversifying strategies, which is not captured in these studies. Thus, the potential link between expanded forage options, profitability, and long-term sustainable dairy farming requires further investigation.

Research on the dietary preferences of pastured dairy cows exhibits considerable variability in study design and features, making cross-study comparisons challenging. Although there is evidence of positive environmental and production outcomes associated with pasture diversification, a deeper understanding of dietary preference in the context of variable external factors that influence grazing behavior is needed. Environmental and management differences across studies, which may or may not be reported, include region and climatic conditions, the specific combinations of plant species sown, baseline soil quality, grazing system, topographical land characteristics, stocking density, and animal access to shade and water. Although the included studies detailed the specific forages present, pasture diversification alone is not sufficient for outcome measurement assessment; pasture functional diversity may be more significant than pasture hyperdiversity (Soder et al., 2007). Furthermore, to assess the maximum potential benefits of pasture diversification, the introduction of diverse plant species must be paired with dynamic land-use strategies that consider the complex interaction between cattle and the land they graze.

Internal factors that influence grazing behavior, such as individual cow differences, may also complicate the interpretation of studies and hinder cross-study comparisons. These individual differences, including early life experiences, herd social dynamics, life stage, and personality, must be accounted for in successful, diversified pasture systems. Early research identified differences in dietary preference between heifers and lactating cows. Within-study comparisons of heifer, lactating, and dry cow grazing preferences may help farmers optimize the pairing of appropriate land with specific dairy cattle cohorts. The application of animal behavior—influenced by physiological, social, and biopsychological factors—to diversified pasture design and management will help prevent the depletion of highly preferred plant species, predict forage yield, and inform pasture rotation schedules, improving overall land utilization. Collectively, a better understanding of the internal and external factors that influence dietary preferences will advance the integration of animal grazing behavior into land-use strategies.

Although research has investigated the environmental and production outcomes of plant diversification, the associated animal welfare outcomes have been largely neglected. There is substantial evidence that, when provided with the opportunity, cows will choose to eat a mixed diet comprised of a rich assortment of forages. Cows will also work to express dietary preferences (Van Os et al., 2018). Furthermore, research on other species highlights the potential welfare benefits of choice and agency, as reviewed by Englund and Cronin (2023), supporting the hypothesis that broadened dietary options may promote positive welfare states. Studies have investigated pasture access and the affective state of dairy cattle (von Keyserlingk et al., 2017; Crump et al., 2021), but to our knowledge, no studies have examined pasture diversification and affective state. Further investigation is required using approaches such as motivation tests or cognitive bias assessments to better understand how pasture diversity, and the increased agency over the expression of dietary preference it allows, impact the affective states of pastured dairy cattle. While pasture diversity can improve productivity and ecosystem services on marginal lands, it may also offer the dairy industry an opportunity to foster its social license by supporting positive welfare states through expanded forage options.

In addition to agronomic studies needed to optimize forage yields and nutrient value on marginal lands, dietary preference studies will also be important for determining which plant species cows eat and how much, during and after land restoration. Most studies included in this review were conducted on established pastureland and studies on lower quality lands typically feature beef cattle. Dietary preferences may manifest differently on poorer quality lands and evolve as plant diversity increases. Without feed supplementation, low-quality forage on marginal lands may not support the energy requirements of adult dairy cattle, especially those bred for high genetic merit milk production. Although largely beyond the scope of this review, this underscores the role of genetics-by-environment interactions as another consideration in the utilization of marginal land for dairy systems.

Sæther et al. (2006) and Pauler et al. (2020) reported differences in dietary preferences of dairy and dairy-cross breeds of varying production levels. These studies suggest that productivity and the associated metabolic demands may affect dietary decision-making, indicating that data should not be extrapolated from research on cattle bred for beef production. Pasture systems for dairy cattle may require a more careful balance between opportunities to express preferences and the costs associated with pursuing these preferences to avoid excessive energy expenditure, especially during more metabolically demanding life stages. Dairy and beef cattle further differ in their early life experiences. Beef calves are often reared with a dam, whereas most dairy calves in the United States are reared in isolation. Beef calves are also more likely to have early-life exposure to grazing, with the dam serving as an experienced model. These differences in early life experiences that affect social and cognitive development may alter grazing behavior and dietary preference (Neave et al., 2018).

Silvopastoral systems and the associated plant species diversity may be a potential solution for the recruitment of marginal land into productive grazing systems for dairy cattle. Silvopasture involves not only the traditional consideration of grass species presence but also the introduction of woody perennials and forage crops as key components of pasture diversification. There is evidence of the wide range of ecosystem services resulting from marginal land conversion to silvopasture in temperate regions of the United States, including improved soil fertility, water quality, carbon sequestration, and biodiversity (Greene et al., 2023). Within carefully managed systems, cattle grazing can restore these nutritionally depleted lands (Teague and Kreuter, 2020), resulting in a higher quantity and quality of forage. Young heifers under less metabolic stress than adult dairy cattle may effectively graze this land without compromising performance. Furthermore, temporary production losses during land conversion to silvopasture may be offset by revenue-generating opportunities such as timber and tree crop yields (Greene et al., 2023). Long-term studies are needed to provide evidence-based guidance on how to identify prospective land for recruitment and how to maintain dairy cattle productivity and welfare during transitional periods of land conversion. Applying the principles of dairy cattle dietary preferences to research investigating supplemental feed provision, intentional plant species introduction, and continuous versus rotational grazing methods on marginal lands may inform grazing management strategies that support sustainable land-use strategies. However, further empirical evidence of dietary preference on lower quality lands is necessary to determine how strategies for optimizing intensive pasture systems can be modified to support restoration-phase marginal land.

Grazing management systems that reconcile pasture diversification, dairy cattle dietary preferences, and marginal land restoration promise a shared solution to improving sustainable land-use strategies. There is evidence that strategic pasture diversification can enhance land quality while supporting dairy cow performance; however, targeted research on marginal lands is needed to understand how we can optimize dairy cattle welfare and performance during landscape restoration. Defining successful pasture ecosystems further requires leveraging the animal's dietary preferences to maximize the benefits of plant diversification. Harnessing our understanding of both plant communities and grazing animals to support their dynamic interactions provides a gateway to reversing landscape degradation while ensuring food security. As we explore ways to maximize agricultural land-use efficiency, the recruitment of marginal land into dairy cattle grazing systems emerges as a compelling area of research, meriting further investigation.

## References

[bib1] Ahmadzai H., Tutundjian S., Dale D., Brathwaite R., Lidderr P., Selvaraju R., Malhotra A., Boerger V., Elouafi I. (2022).

[bib2] Balocchi O.A., López I.F. (2009). Herbage production, nutritive value and grazing preference of diploid and tetraploid perennial ryegrass cultivars (*Lolium perenne* L.). Chil. J. Agric. Res..

[bib3] Bryant R.H., Miller M.E., Greenwood S.L., Edwards G.R. (2017). Milk yield and nitrogen excretion of dairy cows grazing binary and multispecies pastures. Grass Forage Sci..

[bib4] Carmona-Flores L., Bionaz M., Downing T., Sahin M., Cheng L., Ates S. (2020). Milk production, N partitioning, and methane emissions in dairy cows grazing mixed or spatially separated simple and diverse pastures. Animals (Basel).

[bib5] Carpino S., Licitra G., Van Soest P.J. (2003). Selection of forage species by dairy cattle on complex Sicilian pasture. Anim. Feed Sci. Technol..

[bib6] Chapman D.F., Parsons A.J., Cosgrove G.P., Barker D.J., Marotti D.M., Venning K.J., Rutter S.M., Hill J., Thompson A.N. (2007). Impacts of spatial patterns in pasture on animal grazing behavior, intake, and performance. Crop Sci..

[bib7] Cong W.-F., van Ruijven J., Mommer L., De Deyn G.B., Berendse F., Hoffland E. (2014). Plant species richness promotes soil carbon and nitrogen stocks in grasslands without legumes. J. Ecol..

[bib8] Cosgrove G.P., Anderson C.B., Fletcher R.H. (1995). Do cattle exhibit a preference for white clover?. NZGA: Research and Practice Series.

[bib9] Crump A., Jenkins K., Bethell E.J., Ferris C.P., Kabboush H., Weller J., Arnott G. (2021). Optimism and pasture access in dairy cows. Sci. Rep..

[bib10] Csikós N., Tóth G. (2023). Concepts of agricultural marginal lands and their utilisation: A review. Agric. Syst..

[bib11] Englund M.D., Cronin K.A. (2023). Choice, control, and animal welfare: Definitions and essential inquiries to advance animal welfare science. Front. Vet. Sci..

[bib12] FAO (Food and Agriculture Organization of the United Nations) (2017).

[bib13] Finn J.A., Kirwan L., Connolly J., Sebastià M.T., Helgadottir A., Baadshaug O.H., Bélanger G., Black A., Brophy C., Collins R.P., Čop J., Dalmannsdóttir S., Delgado I., Elgersma A., Fothergill M., Frankow-Lindberg B.E., Ghesquiere A., Golinska B., Golinski P., Grieu P., Gustavsson A.-M., Höglind M., Huguenin-Elie O., Jørgensen M., Kadziuliene Z., Kurki P., Llurba R., Lunnan T., Porqueddu C., Suter M., Thumm U., Lüscher A. (2013). Ecosystem function enhanced by combining four functional types of plant species in intensively managed grassland mixtures: A 3-year continental-scale field experiment. J. Appl. Ecol..

[bib14] Greene H., Kazanski C.E., Kaufman J., Steinberg E., Johnson K., Cook-Patton S.C., Fargione J. (2023). Silvopasture offers climate change mitigation and profit potential for farmers in the eastern United States. Front. Sustain. Food Syst..

[bib15] Hesselmann M., Thorne S., Vitra A., Steiner A.K., Leiber F., Dittmann M.T. (2025). Foraging preferences of dairy cows grazing on contrasted multispecies swards. Vet. Anim. Sci..

[bib16] Horadagoda A., Fulkerson W.J., Nandra K.S., Barchia I.M. (2009). Grazing preferences by dairy cows for 14 forage species. Anim. Prod. Sci..

[bib17] Khatiwada B., Acharya S.N., Larney F.J., Lupwayi N.Z., Smith E.G., Islam M.A., Thomas J.E. (2020). Benefits of mixed grass–legume pastures and pasture rejuvenation using bloat-free legumes in western Canada: A review. Can. J. Plant Sci..

[bib18] Moorby J.M., Evans R.T., Scollan N.D., MacRae J.C., Theodorou M.K. (2006). Increased concentration of water-soluble carbohydrate in perennial ryegrass (*Lolium perenne* L.). Evaluation in dairy cows in early lactation. Grass Forage Sci..

[bib19] Neave H.W., Weary D.M., von Keyserlingk M.A.G. (2018). Review: Individual variability in feeding behaviour of domesticated ruminants. Animal.

[bib20] Oliveira B.A., López I.F., Cranston L.M., Poli C.H.E.C., Kemp P.D., Donaghy D.J., Draganova I., López-Villalobos N. (2024). Animal behaviour and dietary preference of dairy cows grazing binary and diverse pastures under the leaf regrowth stage defoliation criterion. Anim. Feed Sci. Technol..

[bib21] Pauler C.M., Isselstein J., Berard J., Braunbeck T., Schneider M.K. (2020). Grazing allometry: Anatomy, movement, and foraging behavior of three cattle breeds of different productivity. Front. Vet. Sci..

[bib22] Pembleton K.G., Hills J.L., Freeman M.J., McLaren D.K., French M., Rawnsley R.P. (2016). More milk from forage: Milk production, blood metabolites, and forage intake of dairy cows grazing pasture mixtures and spatially adjacent monocultures. J. Dairy Sci..

[bib23] Phillips C.J.C., James N.L., Murray-Evans J.P. (1996). Effect of forage supplements on the incidence of bloat in dairy cows grazing high clover pastures. Vet. Rec..

[bib24] Phy T.S., Provenza F.D. (1998). Sheep fed grain prefer foods and solutions that attenuate acidosis. J. Anim. Sci..

[bib25] Rivero M.J., Balocchi O.L., Neumann F.L., Siebald J.A. (2019). Grazing preference of dairy cows and pasture productivity for different cultivars of perennial ryegrass under contrasting managements. Animals (Basel).

[bib26] Roca-Fernández A.I., Peyraud J.L., Delaby L., Delagarde R. (2016). Pasture intake and milk production of dairy cows rotationally grazing on multi-species swards. Animal.

[bib27] Rutter S.M. (2006). Diet preference for grass and legumes in free-ranging domestic sheep and cattle: Current theory and future application. Appl. Anim. Behav. Sci..

[bib28] Rutter S.M., Orr R.J., Yarrow N.H., Champion R.A. (2004). Dietary preference of dairy heifers grazing ryegrass and white clover, with and without an anti-bloat treatment. Appl. Anim. Behav. Sci..

[bib29] Rutter S.M., Orr R.J., Yarrow N.H., Champion R.A. (2004). Dietary preference of dairy cows grazing ryegrass and white clover. J. Dairy Sci..

[bib30] Sæther N.H., Sickel H., Norderhaug A., Sickel M., Vangen O. (2006). Plant and vegetation preferences for a high and a moderate yielding Norwegian dairy cattle breed grazing semi-natural mountain pastures. Anim. Res..

[bib31] Sanderson M.A., Soder K.J., Muller L.D., Klement K.D., Skinner R.H., Goslee S.C. (2005). Forage mixture productivity and botanical composition in pastures grazed by dairy cattle. Agron. J..

[bib32] Smit H.J., Tamminga S., Elgersma A. (2006). Dairy cattle grazing preference among six cultivars of perennial ryegrass. Agron. J..

[bib33] Soder K.J., Rook A.J., Sanderson M.A., Goslee S.C. (2007). Interaction of plant species diversity on grazing behavior and performance of livestock grazing temperate region pastures. Crop Sci..

[bib34] Soder K.J., Sanderson M.A., Stack J.L., Muller L.D. (2006). Intake and performance of lactating cows grazing diverse forage mixtures. J. Dairy Sci..

[bib35] Teague R., Kreuter U. (2020). Managing grazing to restore soil health, ecosystem function, and ecosystem services. Front. Sustain. Food Syst..

[bib36] Totty V.K., Greenwood S.L., Bryant R.H., Edwards G.R. (2013). Nitrogen partitioning and milk production of dairy cows grazing simple and diverse pastures. J. Dairy Sci..

[bib37] Van Os J.M., Mintline E.M., DeVries T.J., Tucker C.B. (2018). Domestic cattle (*Bos taurus taurus*) are motivated to obtain forage and demonstrate contrafreeloading. PLoS One.

[bib38] Vandermeulen S., Ramírez-Restrepo C.A., Marche C., Decruyenaere V., Beckers Y., Bindelle J. (2018). Behaviour and browse species selectivity of heifers grazing in a temperate silvopastoral system. Agrofor. Syst..

[bib39] Villalba J.J., Provenza F.D. (2007). Self-medication and homeostatic behaviour in herbivores: learning about the benefits of nature's pharmacy. Animal.

[bib40] von Keyserlingk M.A., Amorim Cestari A., Franks B., Fregonesi J.A., Weary D.M. (2017). Dairy cows value access to pasture as highly as fresh feed. Sci. Rep..

